# A clinical and molecular study of artesunate + sulphadoxine-pyrimethamine in three districts of central and eastern India

**DOI:** 10.1186/1475-2875-12-247

**Published:** 2013-07-17

**Authors:** Prakriti Srivastava, Jagnyeswar Ratha, Naman K Shah, Neelima Mishra, Anupkumar R Anvikar, Surya K Sharma, Manoj K Das, Bina Srivastava, Neena Valecha

**Affiliations:** 1 National Institute of Malaria Research, Sector 8, Dwarka, New Delhi 110077, India; 2 School of life Sciences, Sambalpur University, Odisha 768019, India; 3 National Institute of Malaria Research, Ranchi Field Unit, Ranchi, Jharkhand, India

**Keywords:** Artesunate + sulphadoxine-pyrimethamine, Plasmodium falciparum, Dihydrofolate reductase, Dihydropteroate synthetase, Falciparum malaria

## Abstract

**Background:**

Artesunate + sulphadoxine-pyrimethamine (AS + SP) is recommended throughout India as the first-line treatment for uncomplicated falciparum malaria. Due to the presence of several eco-epidemiological zones of malaria and variable drug pressure, it is necessary to evaluate the efficacy of this combination in different regions of India. The objective of this study was to use clinical and molecular methods to monitor the efficacy of AS + SP in three diverse sites.

**Methods:**

The study was undertaken in three high endemic sites of central and eastern India. Patients with uncomplicated falciparum malaria were enrolled and followed for 28 days. Molecular genotyping was conducted for merozoite surface protein (*msp1* and *msp2*) to differentiate between re-infection and recrudescence and for the *dhfr* and *dhps* genes to monitor antifolate drug resistance.

**Results:**

In all, 149 patients were enrolled at the three sites. The crude cure rates were 95.9%, 100%, and 100% in Ranchi, Keonjhar, and West Garo Hills respectively. PCR-corrected cure rates were 100% at all sites. In *dhfr*, 27% of isolates had triple mutations, while 46% isolates were double-mutants. The most prevalent mutation was S108N followed by C59R. 164 L mutation was observed in 43/126 (34%) isolates. In *dhps*, most (76%) of the isolates were wild-type. Only 2.5% (2/80) isolates showed double mutation. *dhfr*-*dhps* two locus mutation were observed in 16% (13/80) isolates. Parasite clearance time was not related with antifolate mutations.

**Conclusions:**

AS + SP combination therapy remained effective against falciparum malaria despite common mutations promoting resistance to antifolate drugs. Although the prevalence of double and triple mutations in *dhfr* was high, the prevalence of *dhfr*-*dhps* two locus mutations were low. Even isolates with *dhfr* triple and dhfr-dhps two locus mutations achieved adequate clinical and parasitological response.

## Background

Anti-malarial drug resistance has been a major obstacle in the fight against malaria and has been documented globally in *Plasmodium falciparum.* In India, chloroquine (CQ) resistance was first reported in Assam in 1973 [1] and thereafter several reports of resistance were confirmed from all over India [2-6]. Sulphadoxine-pyrimethamine (SP) became the alternative to CQ for the treatment and control of uncomplicated malaria. It was effective, affordable and easy to comply with, but in course of time the accumulations of mutations in parasite genes coding for two enzymes dihydrofolate reductase (DHFR) and dihydropteroate synthetase (DHPS) led to SP resistance in certain sites, mostly in north-east India [7]. In 2001, the World Health Organization (WHO) recommended the use of artemisinin based combinations therapy (ACT), in which a long-acting partner is added to artemisinin to make it more effective, as first-line treatment for uncomplicated falciparum malaria [8]. Since 2005, most malaria-endemic countries adopted ACT. In 2005, AS + SP was introduced in the National Drug Policy for malaria in India in selected areas, and in 2010 it became the universal first-line drug for falciparum malaria. With the replacement of monotherapy by ACT, it is important to understand the relationship between molecular markers, parasite resistance and treatment failure.

Several challenges exist in understanding this correlation since molecular markers for artemisinin resistance have not been established. These markers however, are well established for SP. Sulphadoxine acts as competitive inhibitor in folate biosynthetic pathway of parasite by inhibiting the enzyme DHPS [9]. Pyrimethamine inhibits the DHFR enzyme of *P. falciparum*, also a component of the folate-biosynthetic pathway. Mutations in some of the key amino acids of this enzyme lead to reduced binding affinity with the drug, leading to resistance [10]. In the AS + SP combination, AS rapidly clears parasitaemia. SP kills the remaining parasites, since it has a long half-life in blood. Thus, for clinical and parasitological treatment outcomes, early treatment failure (ETF) is more likely associated with AS failure and late treatment failure (LTF) with SP failure. The determination of parasite density on day 0 and the proportion of patients positive on day 3 could thus be a key indicator for the *in vivo* susceptibility of artemisinin [11].

Since pre-existing resistance to SP exists in India, especially along the border with Myanmar where reduced artemisinin sensitivity has been reported, it is important to monitor both *in vivo* and *in vitro* responses to this combination. Therefore, the objective of the present study was to: 1) measure the *in vivo* efficacy of AS + SP including treatment failure and parasite clearance; 2) examine molecular markers for SP to understand the mutation pattern; and, 3) correlate molecular marker results with clinical outcomes.

## Methods

This was a single arm prospective study carried out from 2007 to 2010 (Aug-Oct 2007 in Ranchi, Sep-Nov 2007 in Keonjhar and June-Oct 2010 in West Garo Hills) to evaluate the parasitological and clinical response to directly observed treatment for uncomplicated falciparum malaria [12].

### Study sites

**Figure 1:**
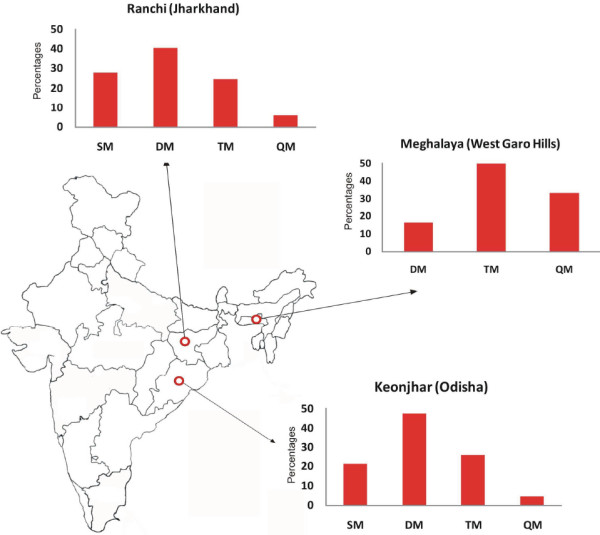
**Selected study sites for AS + SP therapeutic efficacy studies, Keonjhar (Odisha), Ranchi (Jharkhand) and West Garo Hills (Meghalaya) and total mutations in *dhfr *and *dhps *gene, 2007 and 2010. **Abbreviations: SM, single mutation, DM, double mutation, TM, triple mutation, QM, quadruple mutation.

The study was carried out at three sites (Figure 1). Ranchi (Jharkhand): Jharkhand contributes to 7% of the two million reported cases of malaria in India. The study district Ranchi is the capital of Jharkhand and is highly endemic for malaria with an average elevation 629 m above sea level. Ranchi has a hilly topography with dense tropical forests. The main vector of malaria in Ranchi is *Anopheles fluviatilis* and predominant tribes living there are Oram and Munda. Keonjhar (Odisha). In India, Odisha state contributes to 40% of reported *P*. *falciparum* cases. The climate of the study district is hot in summer with high humidity. The district is covered by 30% of forest of the total geographical area. Main vector of malaria is *An. fluviatilis*. There are 16 tribal populations in Keonjhar with the Oram and Munda as predominant tribes. West Garo Hills (Meghalaya): *P*. *falciparum*-related deaths are routinely reported annually from West Garo Hills district, located at the western most part of Meghalaya. There is heavy rainfall during May to September with a seasonal peak from May to July. The main vector of malaria is *Anopheles minimus*[13]. The Khasis are the largest group of tribes, followed by the Garos. Partial data from this study site was already published [14]. However detailed clinical and molecular data are presented in this paper.

### Enrolment

All patients reporting to a local clinic with complaint of fever were examined for presence of parasites in the blood smear. The demographic information (age, gender, body temperature, body weight) were recorded. Peripheral smear was examined and those positive for *P. falciparum* were enrolled. Informed consent was obtained and case record form was completed for each patient.

### Inclusion and exclusion

Patients aged above six months with mono-infection with *P. falciparum,* with parasitaemia of 1,000 to 100,000/μl, axillary temperature ≥37.5°C or history of fever during previous 24 hours and agreeing to follow-up visits, were included in study. Patients with presence of one or more general danger signs or any sign of severe malaria, presence of mixed infection, severe malnutrition, febrile conditions caused by diseases other than malaria, contra-indications related to anti-malarial drugs used, and pregnancy, were excluded from this study.

### Treatment

AS was given at a target dose of 4 mg/kg daily for three days; SP were given as a single dose equivalent to 25/1.25 mg/kg (IPCA Laboratories Ltd, Mumbai). Agewise dosing was followed rather than weightwise [15]. Patients were observed for 30 min and if they vomited during this period, the first dose was repeated.

### Follow-up

Clinical (temperature, vital symptoms, physical parameters) and parasitological (thick and thin slide) assessment were done on days 0, 1, 2, 3 and 7 and then weekly until day 28. However, patients were advised to return on any day during follow-up if symptoms existed. For defaulters, follow-up was done by visiting the patient at their home or place of work. Patients were advised to use personal protection measures to prevent re-infection.

### Microscopy

Thick and thin blood smears were examined for presence of parasite. Thick blood films were stained with Giemsa and parasite density determined by counting parasites against 200 white blood cells (WBC) and assuming WBC count to be 8,000/μl. The parasite clearance time (PCT) was determined based on a daily blood smear as per the WHO protocol for therapeutic efficacy studies. Finger prick blood samples were collected on Whatman 3 mm filter papers on admission and on the day of re-appearance of parasites after complete anti-malarial treatment during follow-up and unscheduled day.

### Molecular analysis

DNA was isolated from dried blood spot using DNA isolation kit according to manufacturer’s instructions (Qiagen, Valencia, CA, USA). To distinguish re-infection and recrudescence, parasite genotyping for polymorphic loci, merozoite surface proteins (*msp*1 and *msp*2) were conducted according to the published method [16]. Mutation specific PCR were conducted to amplify *dhfr* and *dhps* gene according to method described earlier [17]. The *dhfr* fragment of 720 base pair was amplified first and then mutation specific PCR were carried out for codon 16, 51, 59, 108 and 164. In *dhps*, primary PCR were carried out to obtain a PCR product of 1.33 kb. Nested PCR were performed using two flanking and seven mutation-specific primers to detect point mutations at codon 436, 437, 540, 581 and 613. Molecular markers were analyzed in all D0 samples to get data of the baseline antifolate mutations regardless of follow-up and clinical outcomes.

### Classification of outcomes

Treatment outcomes were classified as early treatment failure (ETF), late clinical failure (LCF), late parasitological failure (LPF), adequate clinical and parasitological response (ACPR) on the basis of an assessment of the parasitological and clinical outcome of anti-malarial treatment according to the latest WHO guidelines [18].

### Data analysis

Computer-based applications prepared by WHO for data management and analysis were used (software package developed by WHO’s Global Malaria Programme for evaluating therapeutic efficacy). Data were entered into WHO software for Kaplan-Meier analysis for calculating cure rates. Results were expressed as the cumulative success rate and the proportion of adequate clinical and parasitological response (ACPR) before and after adjustment by PCR. The primary endpoint of the study was the per protocol (PP)-PCR corrected cure rate. The secondary endpoints included the parasite clearance time (PCT), fever clearance time, percent patients without gametocytes on day 28, and proportion of patients with ETF, LTF and LPF. The sum of *dhfr* and *dhps* mutations were defined as the total number of antifolate mutations. Fisher’s exact test was used to find association between antifolate mutations and parasite clearance.

### Sample size

The study was designed according to WHO therapeutic efficacy protocol which recommends a minimum of 50 patients in a site for appropriate precision (5%) to detect an unknown treatment failure rate with 95% confidence. We aimed to recruit approximately 70 patients in each site in case of loss to follow-up.

### Ethics

The study was approved by Institutional Ethical Committee of National Institute of Malaria Research (NIMR). Patients were included in the study only if they or their parents or guardians gave informed consent. All information on patients was kept confidential.

## Results

### *In vivo* efficacy of AS + SP

**Figure 2:**
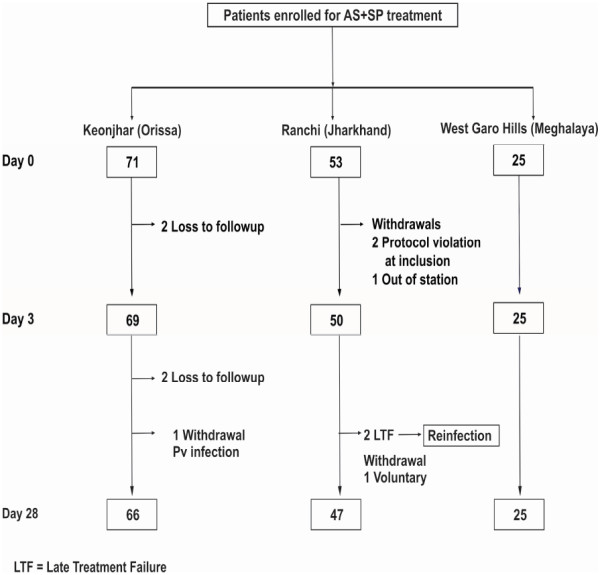
**Flow chart of patients enrolled for AS + SP treatment at Keonjhar (Odisha), Ranchi (Jharkhand) and West Garo Hills (Meghalaya), India, 2007 and 2010. **Abbreviations: Pv, *Plasmodium vivax*; LTF, late treatment failure.

A total of 149 patients were enrolled during 2007–2010 at three sites. Most patients were below 15 years of age with fever on enrolment and parasitaemia between 5,000-50,000/μl blood (Table 1). Out of 149 patients, four (3%) were lost to follow-up and five (3%) were withdrawals (four protocol violations in inclusion and one with *Plasmodium vivax* infection). There were two treatment failures on day 26 and 27 from Ranchi. No clinical or parasitological failures were reported from other sites (Figure 2).

**Table 1:** Demographic and clinical characteristics of patients enrolled for AS + SP therapeutic efficacy studies, central and eastern India, 2007 and 2010

	**Keonjhar (N = 71)**		**Ranchi (N = 53)**		**West Garo Hills (N = 25)**	
	**n**	**%**	**n**	**%**	**n**	**%**
**Gender**						
Male	31	44	22	42	15	60
Female	40	56	31	58	10	40
**Age groups**						
<5	31	44	12	22	7	28
5-14	25	35	19	36	14	56
≥15	15	21	22	42	4	16
**Parasitaemia/μL on day 0**						
<5000	27	38	8	15	12	48
5000-50000	29	41	34	64	10	40
>50000	15	21	11	21	3	12
**Parasite clearance time**						
<24 hours	46	65	48	90	22	98
24-48	23	32	5	10	3	2
>48	2	3				
**Fever on day 0 **(auxiliary temperature) ≥37.5°C	71	100	45	85	8	32
≤37.5°C	0	0	8	15	17	68

Abbreviations: N, total number of patients enrolled; n, out of total enrolled.

The parasite clearance was rapid at all sites and complete clearance of parasitaemia within 48 hours of administration of drug were observed in 97% (69/71) in Keonjhar, 100% (53/53) in Ranchi and (25/25) West Garo Hills (Table 1). Proportion of patients with gametocytes at enrolment was 13% (9/71), 30% (16/53) and 12% (3/25) respectively in Keonjhar, Ranchi and West Garo Hills. Gametocytes were detected in only one patient on day 28 from West Garo Hills.

Out of two cases of treatment failure, one had temperature 38.5°C and 9% of day 0 parasitaemia on day 26 and classed as LCF. Another was classed as LPF as he had temperature 36.8°C and 21% of day 0 parasitaemia on day 28. Molecular genotyping for *msp*1 and *msp*2 of failure samples indicate multiple infections and classified as a re-infection. PP-PCR-uncorrected cure rates were 95.9% (95% CI: 86, 99.5), 100% (95% CI: 94.6, 100), and 100% (95% CI: 86.3, 100) respectively at Ranchi, Keonjhar and West Garo Hills. After PCR-correction, cure rates were 100% at all sites.

### Molecular analysis of *dhfr* and *dhps* gene

All isolates were analysed for *dhfr* mutations encoding pyrimethamine resistance and *dhps* mutations associated with sulphadoxine resistance (Table 2). In *dhfr*, 136 (91%) isolates were successfully amplified. The S108N mutation was the most common and found in all amplified samples. Seventy-one percent of the samples were mutant at amino acid position C59R; I164L mutation was observed in 34% of isolates. In *dhfr*, double mutants (S108**N +** C59**R** or I164**L**) were the most common in all three sites: Ranchi (47%, n = 22/47) and Keonjhar (49%, n = 27/55) and West Garo Hills (17%, n = 3/18). Other than double mutations, the triple mutant C59**R** + S108**N** + I164**L** were also detected in all sites but its prevalence was highest in West Garo Hills (83%, n = 15/18) followed by Ranchi (28%, n = 13/47) and Keonjhar (18%, n = 10/55). Single mutation S108**N** were observed in Keonjhar (33%, n = 18/55) and Ranchi (26%, n = 12/47) only.

**Table 2:** **Prevalence of point mutation in *dhfr *and *dhps *gene in day 0 samples of patients enrolled for AS + SP therapeutic efficacy studies, central and eastern India, 2007 and 2010**

	**Keonjhar (n = 71)**			**Ranchi (n = 53)**		**WGH (n = 25)**	
**Genes**	**Codons**	**n M**	**%M**	**n M**	**% M**	**n M**	**%M**
							
***dhfr***	C59R	59 38	64	53 36	68	18 18	100
	S108N	60 60	100	50 50	100	19 19	100
	I164L	60 20	33	52 18	35	14 5	36
***dhps***	K540E	28 11	39	28 4	12	10 6	60
	A581G	28 8	28	28 5	14	15 0	0

Abbreviations: *WGH*, West Garo Hills; *n*, number; *M*, mutant.

In *dhps*, out of 149 collected isolates, 88 (59%) were successfully amplified. Mutations were found in 19% (n = 17/88) of the samples. A single mutation at amino acid position 581 was observed in 8% (n = 7/88) and at amino acid position 540 was present in 6% (n = 5/88) of isolates. Double mutations at position 540 and 581 were observed in 1% (n = 1/88) of isolates.

Considering *dhfr* and *dhps* two locus point mutations, quadruple mutations were present in 7% (6/80) of isolates and triple mutations in 27% (22/80). The majority 42% (34/80) of isolates had double mutations, while 22% (18/80) of isolates had single mutations. Notably, none of the isolates had quintuple mutations. Twenty-five percent (20/80) of isolates with any of these mutations cleared parasitaemia after 24 hours of introduction of drug (Table 3). Ten different haplotypes were detected, of which, two were present in each site, two in Keonjhar and Ranchi, one (10%) in Ranchi and West Garo Hills, while two (20%) in Ranchi alone and three (30%) in Keonjhar alone. Maximum numbers of haplotype were seen in Keonjhar (Table 4).

**Table 3:** **Antifolate (*dhfr *and *dhps*) mutations, clinical outcome and parasite clearance time of patients enrolled for AS + SP treatment, central and eastern India, 2007 and 2010**

**Type of mutation**	**n(80)**	**PCT >24 h**		**Outcome**
		**n**	**%**	
Quadruple	6	2	34	ACPR
Triple	22	6	27	ACPR
Double	34	7	20	ACPR
Single	18	5	28	ACPR

Abbreviations: *ACPR*, adequate clinical and parasitological response.

**Table 4:** **Prevalence of *dhfr*/*dhps *haplotype in day 0 samples of patients enrolled for AS + SP therapeutic efficacy studies, central and eastern India, 2007 and 2010**

**Mutations**	**Genotypes ( *dhfr */ *dhps *)**	**Keonjhar**		**Ranchi**		**WGH**	
		**(n = 42)**		**(n = 32)**		**(n = 6)**	
		**n**	**%**	**n**	**%**	**n**	**%**
4	N_51_**R**_59_**N**_108_I_164_/S_436_A_437_**E**_540_**G**_581_A_613_	-	-	2	6	-	-
4	N_51_**R**_59_**N**_108_**L**_164_/S_436_A_437_**E**_540_A_581_A_613_	2	4	-	-	2	33
3	N_51_**R**_59_**N**_108_**L**_164_/ S_436_A_437_ K_540_A_581_A_613_	6	14	4	13	-	-
3	N_51_**R**_59_**N**_**108**_I_164_/S_436_A_437_**E**_540_A_581_A_613_	4	10	2	6	3	50
3	N_51_C_59_**N**_108_**L**_164_/S_436_A_437_**E**_540_A_581_A_613_	1	2	-	-	-	-
3	N_51_**R**_59_**N**_108_I_164_/S_436_A_437_K_540_**G**_581_A_613_	-	-	2	6	-	-
2	N_51_**R**_59_**N**_108_I_164_/S_436_A_437_K_540_A_581_A_613_	12	29	13	41	1	17
2	N_51_C_59_**N**_108_**L**_164_/S_436_A_437_K_540_A_581_A_613_	4	10	-	-	-	-
2	N_51_C_59_**N**_108_I_164_/S_436_A_437_**E**_540_A_581_A_613_	4	10	-	-	-	-
1	N_51_C_59_**N**_108_I_164_/S_436_A_437_K_540_A_581_A_613_	9	21	9	28	-	-

Abbreviations: *WGH*, West Garo Hills.

Mutated amino acids are in bold.

### Correlation of molecular markers with clinical outcomes

Among the two treatment failures, molecular genotyping of isolates of day 0 and failure day revealed that in both cases isolates had double mutations in *dhfr* (108 + 59) in day 0, while failure day isolates had an additional mutation in *dhps*, one with 540, the other with 581 mutations.

Complete clearance of parasitaemia in less than 24 hours were observed in 78% (116/149) of patients, while 21% (31/149) cleared in between 24 to 48 hours. Only 1% (2/149) cleared in more than 48 hours. There was no statistically significant association between parasite clearance time and increased mutations in *dhfr* and *dhps* (*P* value = 1.24). However, among the isolates that cleared parasitaemia in more than 48 hours, all of them carried 108 + 59 mutations. Isolates with 108 + 164 + 540 cleared parasites between 24 to 48 hours.

## Discussion

AS + SP remained efficacious for the treatment of *P. falciparum* across diverse sites in central and eastern India. Molecular genotyping of *dhfr* and *dhps* genes indicate the dominance of isolates with *dhfr* double mutation and *dhps* wild type suggesting the ongoing development of antifolate resistance.

Despite the presence of mutations in *dhfr* and *dhps* gene, AS + SP treatment remained successful. This may be due to several reasons: first, the action of artemisinin in combination therapy, which kills parasites rapidly and reduces SP drug pressure on the parasite, preserving the latter’s efficacy; second, the rarity of *dhfr*-*dhps* quintuple mutations, which are virtually synonymous with SP treatment failure, in the population studied. However, it is likely that in the course of time such haplotypes may develop and spread in the face of sustained selection pressure; third, host immunity contributes to treatment efficacy and the study population was dominated by persons of tribal ethnicity living in highly endemic areas, who would have high immunity. The findings of this study were similar to those of Mishra *et al.* who reported AS + SP combination as a safe and effective treatment for uncomplicated falciparum malaria in India during 2009–2010 across 22 sites [14]. This study contributes ‘baseline’ data on antifolate resistance as the bulk of samples were collected in 2007 prior to the widespread scale-up of AS + SP.

SP is extensively used, widely available, inexpensive, and slowly eliminated from the body. These factors have promoted the emergence and spread of SP-resistant parasites. Mutations in *dhfr* gene are related to pyrimethamine resistance and develop stepwise, starting with the mutation at codon S108N (Ser-Asn), followed by subsequent mutations at codon 59 (Cys-Arg), 51 (Asn-Ile) and 164 (Ile-Leu). In India, the most common *dhfr* mutation is S108N, followed by C59R, and N51I [17,19,20]. The results of this study showed a similar pattern with the exception of an absence of mutation in codon N51I. It was observed the I164L mutation associated with high levels of pyrimethamine resistance [21]. Previous studies in Assam, in north-east India and Odisha in eastern India, documented the *dhfr* triple mutant Asn108 + Ile51 + Arg59 associated with pyrimethamine resistance [17]. These combinations of mutations were not detected in the present study sites which are in nearby states. The *dhfr* double mutations (S108N + C59R) were the most prevalent here similar to other reported results [22]. This study did not detect any quadruple mutations in *dhfr* such as those reported from the Andaman and Nicobar Islands by Ahmed [20] and Das *et al.*[23]. The success rate of *dhps* gene amplification was low compared to amplification of the *dhfr* gene; however, isolates where it was successfully amplified were wild type. Other authors have also reported the dominance of wild type *dhps* genotype in India [14,17,22,]. The prevalence of *dhps* single mutation (K540E or A581G) and double mutations (540 + 580) were low and triple mutant *dhps* alleles were not detected though reports exist, again, from the Andaman and Nicobar Islands [24].

Regional variations of *dhfr*-*dhps* haplotypes were extensive, with some haplotypes common among Jharkhand, Odisha and West Garo Hills, while others were site specific. The highest number of haplotypes was present in Odisha, which contributes the highest proportion of the reported malaria burden in the country. Thus, high transmission generates higher diversity. In low-transmission areas in contrast, self-recombination leads to greater fixation of haplotypes. Ahmed *et al.*[17] described similar regional differences where more diverse genotypes were seen in the higher transmission states of Assam and Odisha. In addition to transmission, drug pressure varies across states. In low-transmission settings a greater proportion of infections are likely to be symptomatic, and since these areas also tend to be better developed, more cases will have access to care leading to high selection pressure. Anderson *et al.*[25] suggest resistance to SP appears to arise *de novo* much less frequently but spreads rapidly since gametocyte production is stimulated by this drug. High-level pyrimethamine-resistant alleles have emerged in independent foci from which they have rapidly spread to the nearby areas. In contrast, Vinayak *et al.*[26] found single origin for *dhfr*-resistant alleles in the Thai-Cambodia region, while *dhps*-resistant alleles had multiple origins. Lumb *et al.*[27] reported several unique haplotypes among mutant *dhps* alleles and concluded that the *dhps* alleles in India have evolved from multiple genetic backgrounds.

Delayed parasite clearance time is the most important signal for artemisinin resistance [28]. The parasite clearance was rapid at all sites in this study. This rapid clearance of parasitaemia is suggestive of continued artemisinin effectiveness in India though better inference could be made with precise data on parasite clearance rates. No statistically significant association between parasite clearance time and increased mutations in *dhfr* and *dhps* was observed, which was not surprising if the artemisinin component drives clearance. Since the treatment efficacy was high, it was not possible to correlate molecular results with subsequent treatment failure.

The present study had several limitations. Foremost was a follow-up of only 28 days. As the partner drug (SP) has a long half-life, extended follow-up for 42 days could have detected potential late treatment failures emerging with the elimination of residual drug levels. Second, only 25 patents were enrolled in West Garo Hills because enough eligible patients were not found at the recruitment site during the transmission season. Third, the lack of treatment failures restricted the ability to determine the relationship between molecular markers and AS + SP failure. Fourth, the parasite clearance time (PCT) was determined based on a daily blood smear. Finally, while three sites provided some geographic coverage, the results may not be generalizable across India given the size of the country and diversity of malaria ecotypes. The diversity of findings in *dhfr* and *dhps* mutations alone suggests the need for routine monitoring especially in locations with a high prevalence of markers associated with anti-malarial drug resistance. One potential solution, which would minimize the need for expensive *in vivo* trials, is the targeting of such studies using molecular surveillance [29].

## Conclusion

This study suggests that AS + SP remains effective for the treatment of uncomplicated falciparum malaria in central and eastern India despite the presence of mutations in the *dhfr* and *dhps* genes. Regular monitoring of the efficacy of AS + SP will help safeguard this combination treatment. Molecular studies of *dhfr* and *dhps* would be helpful in detecting increased resistance against the partner drug SP and potentially targeting study sites to where treatment failure is likely to emerge.

## Abbreviations

ACTs: Artemisinin combination therapy; AS + SP: Artesunate + sulphadoxine-pyrimethamine; Pf: *Plasmodium falciparum*; dhfr: Dihydrofolate reductase; dhps: Dihydropteroate synthetase; PCT: Parasite clearance time.

## Competing interests

The authors have declared that they have no competing interests.

## Authors’ contributions

PS: molecular studies and preparation of manuscript; NKS: review for molecular studies and manuscript; JR, NM and AA: critical review of manuscript; SKS and MKS: supervision and co-ordination of field work; BS: parasitological assessments; NV: development of protocol, quality control and review of manuscript. The authors have read and approved the final manuscript.

## References

[B1] Sharma VP Battling the malaria iceberg with chloroquine in India Malar J 2007 6 105 10.1186/1475-2875-6-105 17683630 PMC1995206

[B2] Seghal P Sharma M Sharma S Resistance to chloroquine in falciparum malaria in Assam state, India J Commun Dis 1973 5 175 180

[B3] Barkakaty BN Narasimham MV Problem of anti malarial drug resistance in *Plasmodium falciparum* in Mizoram Indian J Malariol 1992 29 89 93 1459310

[B4] Dua VK Kar PK Kumar S Sharma VP In vivo and In vitro sensitivity of *Plasmodium falciparum* to chloroquine at Indian Oil Corporation, Mathura, India Indian J Malariol 1993 30 29 35 8319813

[B5] Giri A Das MK Response of *P*. *falciparum* to chloroquine in Car Nicobar Island Indian J Malariol 1994 31 27 30 7958126

[B6] Sathpathy SK Jena RC Sharma RS Sharma RC Status of *Plasmodium falciparum* resistance to chloroquine in Orissa J Commun Dis 1997 26 145 151 9282514

[B7] Biswas S Escalante A Chaiyaroj S Anglasekmine P Lal AA Prevalence of point mutation in the dihydrofolate reductase and dihydropteroate synthetase genes of *Plasmodium falciparum* isolates from India and Thailand: a molecular epidemiologic study Trop Med Int Health 2000 5 737 743 10.1046/j.1365-3156.2000.00632.x 11044269

[B8] WHO Guidelines for the treatment of malaria 2006 Geneva: World Health Organization

[B9] Brown GM The biosynthesis of folic acid II. Inhibition by sulfonamides J Biol Chem 1962 237 530 540 13873645

[B10] Le Bras J Durand R The mechanism of resistance to antimalarial drugs in *Plasmodium falciparum* Fundam Clin Pharmacol 2003 17 147 153 10.1046/j.1472-8206.2003.00164.x 12667224

[B11] Dondrop AM Nosten F Yi P Das D Tarning APP Lwin KM Ariey F Hanpithakpong W Lee SJ Ringwald P Silamut K Imwong M Chotivanich K Lim P Herdman T Sen Sam A Yeung S Singhasivanon P Day NPJ Lindegardh N Socheat D White NJ Artemisinin resistance in *Plasmodium falciparum* malaria N Engl J Med 2009 361 455 467 10.1056/NEJMoa0808859 19641202 PMC3495232

[B12] World Health Organization Assessment and monitoring of antimalarial drug efficacy for the treatment of uncomplicated falciparum malaria 2003 http://whqlibdoc.who.int/hq/2003/WHO_HTM_RBM_2003.50.pdf

[B13] Vas D Sangma BM Dash AP Persistent transmission of malaria in Garo Hills of Meghalaya bordering Bangladesh, north-east India Malar J 2010 9 263 10.1186/1475-2875-9-263 20858290 PMC2955675

[B14] Mishra N Singh JPN Srivastava B Arora U Shah NK Ghosh SK Bhatt RM Sharma SK Das MK Kumar A Anvikar AR Kaitholia K Gupta R Sonal JS Dhariwal AC Valecha N Monitoring antimalarial drug resistance in India via sentinel sites: outcomes and risk factors for treatment failure, 2009–2010 Bull World Health Organ 2012 90 895 904 23284195 10.2471/BLT.12.109124 PMC3524963

[B15] PMID Revised National Drug Policy for treatment of malaria J Indian Med Assoc 2010 108 844 845 21661462 21661462

[B16] Snounou G Zhu X Siripoon N Jarra W Thaithong S Brown KN Viriyakosol S Biased distribution of msp1 and msp2 allelic variants in *Plasmodium falciparum* populations in Thailand Trans R Soc Trop Med Hyg 1999 93 369 374 10.1016/S0035-9203(99)90120-7 10674079

[B17] Ahmed A Bararia D Vinayak S Yameen M Biswas S Dev V Kumar A Ansari AM Sharma YD *Plasmodium falciparum* isolates in India exhibit a progressive increase in mutations associated with sulfadoxine-pyrimethamine resistance Antimicrob Agents Chemother 2004 48 879 889 10.1128/AAC.48.3.879-889.2004 14982779 PMC353157

[B18] World Health Organization Methods for surveillance of antimalarial drug efficacy 2009 2009 http://whqlibdoc.who.int/publications/2009/9789241597531_eng.pdf

[B19] Sharma YD Genetic alteration in drug resistance markers of *Plasmodium falciparum* Indian J Med Res 2005 121 13 22 15713974

[B20] Ahmed A Das MK Dev V Saifi MA Khan W Sharma YD Quadruple mutations in dihydrofolate reductase of *Plasmodium falciparum* isolates from Car Nicobar Island India. Antimicrob. Agents Chemother 2006 50 1546 1549 10.1128/AAC.50.4.1546-1549.2006 PMC1426982 16569880

[B21] Andriantsoanirina V Durand R Pradines B Baret E Bouchier C Ratsimbasoa A In vitro susceptibility to pyrimethamine of DHFR I164L single mutant *Plasmodium falciparum* Malar J 2011 10 283 10.1186/1475-2875-10-283 21951962 PMC3192713

[B22] Garg S Saxena V Kanchan S Sharma P Mahajan S Kochar D Das A Novel point mutations in sulfadoxine resistance genes of *Plasmodium falciparum* from India Acta Trop 2009 110 75 79 10.1016/j.actatropica.2009.01.009 19283899

[B23] Das MK Lumb V Mittra P Singh SS Dash AP Sharma YD High chloroquine treatment failure rates and predominance of mutant genotypes associated with chloroquine and antifolate resistance among falciparum malaria patients from the island of Car Nicobar, India J Antimicrob Chemother 2010 65 1258 1261 10.1093/jac/dkq090 20363804

[B24] Lumb V Das MK Mittra P Ahmed A Kumar M Kaur P Dash AP Singh SS Sharma YD Emergence of an unusual sulfadoxine-pyrimethamine resistance pattern and a novel K540N mutation in dihydropteroate synthetase in *Plasmodium falciparum* isolates obtained from Car Nicobar Island, India, after the 2004 Tsunami J Infect Dis 2009 199 1064 1073 10.1086/597206 19220141

[B25] Anderson T Mapping the spread of malaria drug resistance PLoS Med 2009 6 e1000054 10.1371/journal.pmed.1000054 19365538 PMC2661254

[B26] Vinayak S Alam MT Hayden TM Mc Cllum AM Sem R Shah NK Lim P Muth S Rogerrs WO Fandeur T Barnwell JW Escalante AA Wongsrichanalai C Ariey F Meshnick SR Udhayakumar V Origin and evolution of sulfadoxine resistant *Plasmodium falciparum* PLoS Pathog 2010 6 e1000830 10.1371/journal.ppat.1000830 20360965 PMC2847944

[B27] Lumb V Das MK Singh N Dev V Khan W Sharma YD Multiple origins of *Plasmodium falciparum* dihydropterote synthetase mutant alleles associated with sulfadoxine resistance in India Antimicrob Agents Chemother 2011 55 2813 2817 10.1128/AAC.01151-10 21422213 PMC3101454

[B28] Briena CO Henricha PP Passia N David A Fidocka B Recent clinical and molecular insights into emerging artemisinin resistance in *Plasmodium falciparum* Curr Opin Infect Dis 2011 24 570 577 10.1097/QCO.0b013e32834cd3ed 22001944 PMC3268008

[B29] Shah NK Alker AP Sem R Susanti AI Muth S Maguire JD Duong S Ariey F Meshnick SR Wongsrichanalai C Molecular surveillance for multidrug-resistant *Plasmodium falciparum*, Cambodia Emerg Infect Dis 2008 14 1637 1640 10.3201/eid1410.080080 18826834 PMC2609877

